# Aldehyde Dehydrogenase 2 (ALDH2) in Rat Fatty Liver Cold Ischemia Injury

**DOI:** 10.3390/ijms19092479

**Published:** 2018-08-22

**Authors:** Arnau Panisello-Roselló, Norma Alva, Marta Flores, Alexandre Lopez, Carlos Castro Benítez, Emma Folch-Puy, Anabela Rolo, Carlos Palmeira, René Adam, Teresa Carbonell, Joan Roselló-Catafau

**Affiliations:** 1 Experimental Hepatic Ischemia-Reperfusion Unit, Institut d’Investigacions Biomèdiques de Barcelona (IIBB), Spanish National Research Council (CSIC), 08036 Barcelona, Catalonia, Spain; arnau.panisello.rosello@gmail.com (A.P.-R.); emma.folch@iibb.csic.es (E.F.-P.); 2 Department of Cell Biology, Physiology and Immunology, Faculty of Biology, Universitat de Barcelona, 08028 Barcelona, Catalonia, Spain; nvalva@ub.edu (N.A.); mfloreso10@alumnes.ub.edu (M.F.); 3 Centre Hépato-Biliaire, AP-PH, Hôpital Paul Brousse, 94800 Paris, France; alexandregl.lopez@gmail.com (A.L.); ccastrob@gmail.com (C.C.B.); rene.adam@aphp.fr (R.A.); 4 Center for Neurosscience and Cell Biology, Universidade de Coimbra, 300-370 Coimbra, Portugal; anpiro@ci.uc.pt (A.R.); palmeira@uc.pt (C.P.)

**Keywords:** ALDH2, MDA, 4-hydroxynonenal (4-HNE), caspases 3, apoptosis, IGL-1, UW, HTK

## Abstract

Institut George Lopez-1 (IGL-1) and Histidine-tryptophan-ketoglutarate (HTK) solutions are proposed as alternatives to UW (gold standard) in liver preservation. Their composition differs in terms of the presence/absence of oncotic agents such as HES or PEG, and is decisive for graft conservation before transplantation. This is especially so when fatty (steatotic) livers are used since these grafts are more vulnerable to ischemia insult during conservation. Their composition determines the extent of the subsequent reperfusion injury after transplantation. Aldehyde dehydrogenase-2 (ALDH2), a mitochondrial enzyme, has been reported to play a protective role in warm ischemia-reperfusion injury (IRI), but its potential in fatty liver cold ischemic injury has not yet been investigated. We evaluated the relevance of ALDH2 activity in cold ischemia injury when fatty liver grafts from Zucker Obese rats were preserved in UW, HTK, and IGL-1 solutions, in order to study the mechanisms involved. ALDH2 upregulation was highest in livers preserved in IGL-1. It was accompanied by a decrease in transaminases, apoptosis (Caspase 3 and TUNEL assay), and lipoperoxidation, which was concomitant with the effective clearance of toxic aldehydes such as 4-hydroxy-nonenal. Variations in ATP levels were also determined. The results were consistent with levels of NF-E2 p45-related factor 2 (Nrf2), an antioxidant factor. Here we report for the first time the relevance of mitochondrial ALDH2 in fatty liver cold preservation and suggest that ALDH2 could be considered a potential therapeutic target or regulator in clinical transplantation.

## 1. Introduction

Organ cold storage using commercial preservation solutions is a mandatory step for mitigating cold ischemic injuries and preserving the graft in the best possible conditions for transplantation [1,2]. During a graft’s cold storage, the organ is subjected to hypoxia which provokes an energy metabolism breakdown, lactate generation, and finally cell death and organ failure [3]. This ischemic damage is then exacerbated by the oxygen supply to the organ during the restoration of the blood flow (reperfusion), during which massive amounts of reactive oxygen species (ROS) are generated and extend the graft’s damage in a process known as ischemia reperfusion injury (IRI) [4]. IRI is responsible for organ failure during the first week after transplantation, leaving aside the immunological rejection processes [4,5]. 

The lack of organs available for transplantation has obliged physicians to use marginal grafts such as steatotic specimens, which are highly vulnerable to IRI. This practice increases the incidence of primary failure and finally compromises the transplant outcome [6,7]. So the selection of preservation solution is crucial for liver transplantation [8], especially when fatty liver grafts are used [7].

The organ preservation solutions most widely used for transplantation of abdominal organs are the University of Wisconsin (UW), histidine-tryptophan-ketoglutarate (HTK), and more recently, Institute Georges Lopez (IGL-1) [8]. Briefly, the most important difference between UW, IGL-1, and HTK concerns the presence or absence of oncotic agents such as hydroxyl-ethyl-starch (HES) in UW, and poly-ethylene-glycol 35 (PEG35) in IGL-1, besides other components. In contrast, HTK does not contain any oncotic agents [3,9].

ROS are generated mainly in the mitochondria (especially during reperfusion), leading to the generation of lipoperoxides and other toxic aldehyde forms such as 4-hydroxynonenal (4-HNE) or malondialdehyde (MDA). The compound 4-HNE is a product of lipid peroxidation of membranes such as that of the mitochondria, and may cause cell toxicity [10,11]. The accumulation of 4-HNE reduces membrane integrity; in turn, it inhibits the proteasome function, triggers protein accumulation, inhibits electron transport chain activity, reduces the Krebs cycle activity, increases mitochondrial permeability and causes apoptosis [12,13]. It is well known that MDA also inflicts organelle damage on a wide variety of cell components and cell signaling sensitive pathways, exposing the cell to a high level of stress and finally leading it to its death. Therefore, lipoperoxides and toxic aldehyde adducts (measured as 4-HNE and MDA respectively), are considered suitable markers for oxidative stress processes [11,13]. Furthermore, advanced oxidation protein products (AOPP) have been proposed as non-specific markers of oxidation [14,15]. We, therefore, decided to study an antioxidant factor, the transcription factor NF-E2 p45-related factor 2 (Nrf2), which upregulates antioxidant, cytoprotective and redox homeostasis mechanisms under stress conditions [16]. This factor is also involved in hepatic lipid metabolisms [17].

Alcohol-dehydrogenase 2 (ALDH2) is a mitochondrial enzyme which is ubiquitously expressed in all tissues, with a particularly high presence in the liver, where it is mostly involved in the ethanol metabolism and plays a crucial role in the detoxification of reactive acetaldehydes [18]. More recently, it has been associated with several pathophysiological disorders such as cardiovascular diseases, strokes, diabetes mellitus and aging, and there is increasing evidence of its involvement in IRI [18,19]. Furthermore, ALDH2 has been reported to promote cytoprotective mechanisms in IRI, contributing to the active cleansing of toxic sub-products of oxidation such as 4-HNE [19].

The protective effects of ALDH2 activation on the complex physiopathology of IRI have been investigated in several organs such as the heart [20,21], brain [22], intestine [23], and more recently in liver [24]. However, reports of the action of ALDH2 against hepatic ischemic insult are scarce [24], and nonexistent in cold fatty liver preservation. In view of the current lack of data, we focused our interest on the mitochondrial ALDH2 in cold ischemia injuries (which occurs in cold graft preservation before transplantation) and whether it might be influenced by the different commercial preservation solutions (UW, IGL-1, and HTK), generally used in liver transplantation [8]. We aimed to assess the relevance of ALDH2 in fatty liver preservation and to determine whether its action is modulated by the choice of the preservation solution. This modulation may be crucial for changing the ALDH2/4-HNE balance in order to improve the graft’s protection against cold ischemia injuries during preservation.

## 2. Results

**Figure 1:**
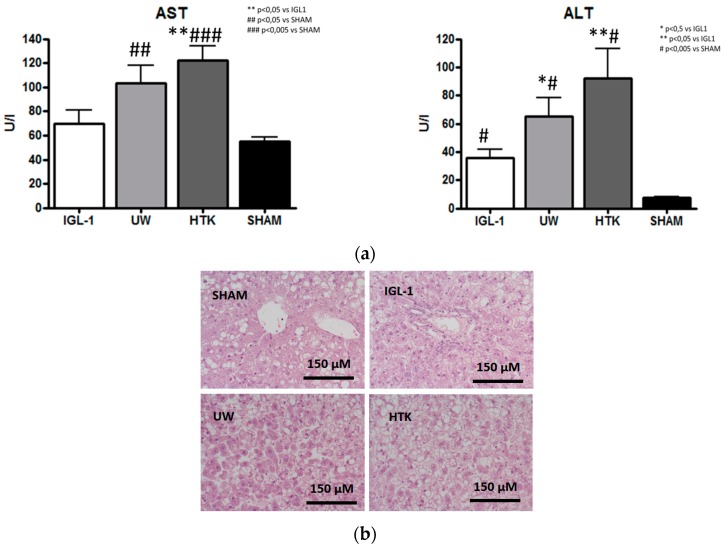
The hepatic injury in fatty liver grafts preserved for 24 h at 4 °C in IGL-1 (Institut George Lopez-1), UW (University of Wisconsin), and HTK (histidine-tryptophan-ketoglutarate), reflected as (**a**) transaminases aspartate aminotransferase (AST) and alanine aminotransferase (ALT); and (**b**) Photomicrographs of livers stained with eosin/hematoxylin corresponding to fatty liver grafts preserved for 24 h at 4 °C in IGL-1, UW, and HTK. Control group showed normal hepatic architecture with macro- and microvesicular fatty infiltration. In IGL-1 group, well-preserved lobular architecture with minimal sinusoidal dilatation and cell swelling were observed, whereas in UW and HTK livers extensive areas of cell dissociation, cell swelling, and disintegration of hepatic architecture were seen. Bar graphs represent mean ± SD, * *p* < 0.05 vs. IGL-1, ** *p* < 0.005 vs. IGL-1 ^#^ *p* < 0.05 vs. SHAM. ^##^ *p* < 0.005 vs. SHAM and ^###^ *p* < 0.005 vs. SHAM.

After 24 h of preservation with the different solutions (IGL-1, UW, HTK) at 4 °C, the liver damage in Zucker Obese rats was measured as alanine aminotransferase and aspartate aminotransferase (ALT/AST) release using the liver effluent wash-out as a sample. Livers preserved in IGL-1 showed lower ALT/AST release than those preserved in UW and HTK (Figure 1a). These results were correlated with the corresponding histological findings using eosin/hematoxylin staining (Figure 1b).

Since the ATP-energy breakdown occurs during ischemia [3], we measured and compared ATP levels in the IGL-1, UW, and HTK preservation solutions. Despite a significant reduction in ATP levels in all groups when compared to the SHAM group (no damage), the IGL-1 solution was the most effective in preventing ATP depletion.

**Figure 2:**
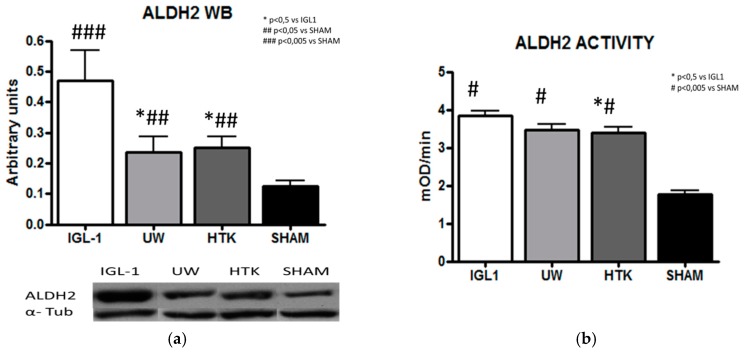
(**a**) represents the ALDH2 protein expression measured with arbitrary units by dividing total amount of protein by total amount of α-tubulin. (**b**) represents ALDH2 activity in fatty liver grafts preserved in UW, IGL-1, and HTK measured as changes of optical density per minute. Bar graphs represent mean ± SD, * *p* < 0.05 vs. IGL-1, ^#^ *p* < 0.05 vs. SHAM. ^##^ *p* < 0.005 vs. SHAM and ^###^ *p* < 0.005 vs. SHAM.

Given that ALDH2 has been shown to promote cytoprotective effects against liver warm IRI insult [24], we next evaluated the amount ALDH2 from liver homogenate (Figure 2a) and its activity (Figure 2b) in cold ischemic preservation. ALDH2 activity levels seemed to be only slightly higher in liver grafts preserved with IGL-1, but the amount of total ALDH2 protein found in the IGL-1 group was almost twice as high as in UW or HTK. This surprising result seems to indicate that there is no correlation between the ALDH2 protein expression and activity. In both cases, the ALDH2 levels in the SHAM group did not differ significantly with regard to the other groups.

**Figure 3:**
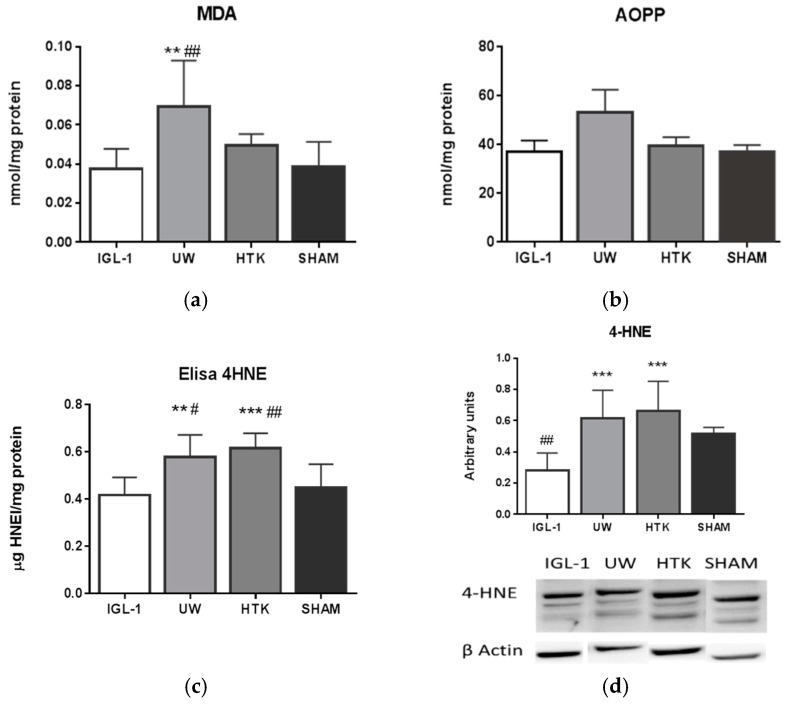
Changes in malondialdehyde (MDA) and in advanced oxidation protein products (AOPP) are measured in (**a**,**b**) respectively. The activity of 4-hydroxynonenal (4-HNE) is measured in (**c**), while its total amount of protein is represented with arbitrary units by dividing total amount of protein by total amount of β-actin in (**d**). Bar graphs represent mean ± SD, ** *p* < 0.005 vs. IGL-1, *** *p* < 0.0005 vs. IGL-1, ^##^ *p* < 0.005 vs. SHAM.

The next step was to explore whether ALDH2 was associated with changes in oxidation sub-products under these conditions. To do so, we measured 4-HNE, MDA, and advanced oxidation protein products (AOPP, a general marker for oxidative stress) from the liver homogenate (Figure 3). As Figure 3 shows, ALDH2 profiles are inversely proportional to changes in 4-HNE levels. However, no correlation between ALDH2 and MDA or AOPP was found, though increased levels of MDA were found in the UW group.

**Figure 4:**
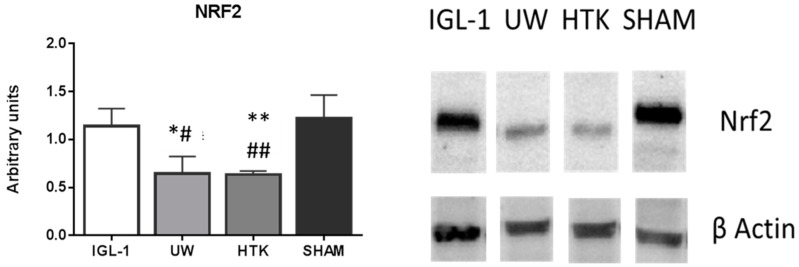
Figure 4 represents the changes in total Nrf2 protein expression divided by total β-actin in liver grafts preserved in UW, IGL-1, and HTK solutions respectively after 24 h of cold storage. Bar graphs represent mean ± SD, * *p* < 0.05 vs. IGL-1, ** *p* < 0.005 vs. IGL-1 ^#^ *p* < 0.05 vs. SHAM. ^##^ *p* < 0.005 vs. SHAM.

We also explored whether this prevention of oxidative stress might be associated with the Nrf2 protein expression from liver homogenate, a well-known antioxidant marker in liver ischemia reperfusion injury [25,26,27]. A significant upregulation was found in the IGL-1 group compared with HTK and UW (Figure 4).

**Figure 5:**
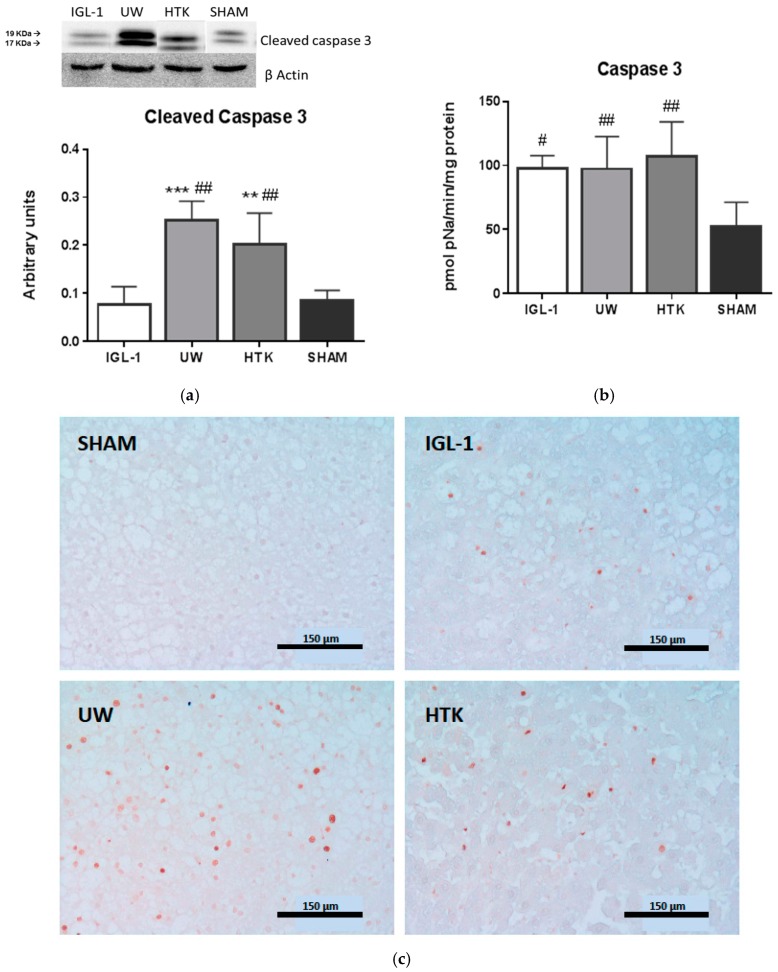
Apoptosis during cold preservation of fatty liver grafts in IGL-1, UW, and HTK is measured as cleaved caspase 3 divided by β-actin (**a**) and caspase 3 activity (**b**). Apoptotic cells analyzed with TUNEL assay are shown in (**c**). Bar graphs represent mean ± SD, ** *p* < 0.005 vs. IGL-1, *** *p* < 0.0005 vs. IGL-1, ^#^ *p* < 0.05 vs. SHAM, ^##^ *p* < 0.005 vs. SHAM.

Finally, given that ALDH2 has been associated with the prevention of liver apoptosis [24], we evaluated the levels of liver apoptosis by measuring cleaved caspase 3 (Casp3) (Figure 5a) and its activity (Figure 5b) in our conditions of cold ischemia graft preservation in the three different solutions. Fatty liver grafts preserved with IGL-1 showed lower levels of caspase 3 (both in the amount of protein and activity). These alterations were confirmed by the corresponding histological findings using TUNEL assay techniques (Figure 5c).

## 3. Discussion

We explored the importance of liver ALDH2 and its possible consequences for cold-preserved fatty liver graft injury prevention, by comparing three well-known preservation solutions that are widely used for abdominal transplantation: UW, IGL-1, and HTK. We were able to establish in our experimental model that the differences found in fatty liver graft preservation were due to the different compositions of the solutions (Table 1).

The outcome of the different degrees of preservation is expressed in the different levels of hepatic damage (i.e, in transaminases and histological findings) in which IGL-1 presented better results than either UW or HTK. These improved preservation conditions were associated with increases in markers such as ALDH2 and Nrf2, and reductions in oxidative parameters. The data reported here reflect the importance of limiting the extent of ischemic damage during cold storage as this defines the extent of the subsequent reperfusion injury after restoring the blood flow to the organ. In this process, the activation of the mitochondrial enzyme was shown to be an important therapeutic target against oxidative damage [19].

In hypoxic conditions (poor/low oxygen input), a situation that arises in cold static preservation, the activity of the metabolic machinery is residual compared to the situation in fully aerobic conditions; however, it continues to function, and these margins of work are the ones that define how the organ will respond to the incoming reperfusion. In this context, the benefits of ALDH2 in cold fatty liver preservation will last beyond the ischemic stage. We showed that reinforced ALDH2 action in graft cold storage with IGL-1 can be considered as a preconditioning tool for reducing the high vulnerability of fatty liver grafts in the setting of IRI. Similarly, with regard to the prevention of hepatic damage (Figure 1), ALT/AST levels in the IGL-1 group were lower than those preserved with UW or HTK (Figure 1a), as confirmed by histological findings (Figure 1b).

**Figure 6:**
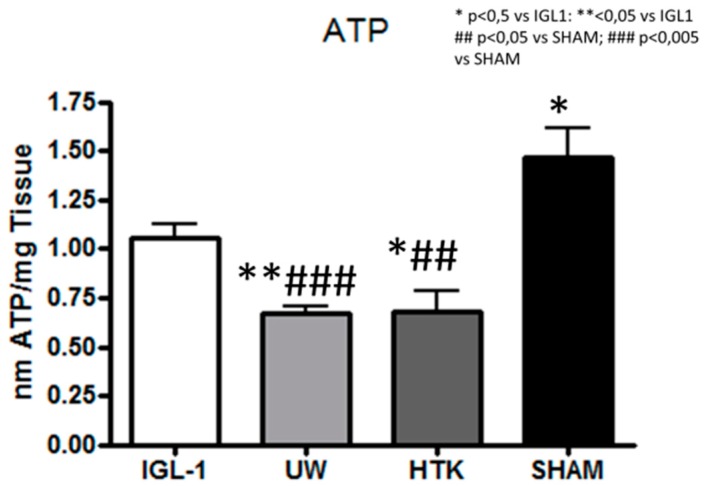
Figure 6 represents ATP levels in fatty liver grafts preserved for 24 h at 4 °C in IGL-1, UW, and HTK measured in nano mols of ATP per milligram of tissue. Bar graphs represent mean ± SD, * *p* < 0.05 vs. IGL-1, ** *p* < 0.005 vs. IGL-1, ^##^ *p* < 0.005 vs. SHAM and ^###^ *p* < 0.005 vs. SHAM.

During the cold ischemic insult, the graft’s capacity to produce ATP is impaired. Later, ROS will open the mitochondrial permeability transition pore, leading to the release of potentially dangerous molecular patterns [28]. In our experimental conditions characterized by prolonged ischemia (24 h at 4 °C), in the absence of ATP or of the capacity to produce it the cell will start upregulating cell-survival mechanisms such as cytoprotective autophagy to cope with the energetic requirements This process is in line with the effective prevention of ATP failure when grafts are conserved in an IGL-1 solution (see Figure 6). During cold ischemia preservation the increasing cytoprotective autophagy protects the graft [29] but during reperfusion, it may be deleterious; the cell becomes apoptotic and dies [30]. These events during an ischemic graft conservation in contrast with those that occur in aerobic conditions, in which liver mitochondria are the main source of ROS in the cell. This situation is especially relevant for fatty livers, which are more vulnerable to oxidation when they are used for transplantation purposes [31].

In our experimental conditions, we stress that at low temperatures (4 °C) the metabolism is reduced by 5 to 10%: rates at which the cells can still retain vital metabolic functions. However, after prolonged hypoxia, and over time, hypothermia may lead to liver injury induced by sodium/potassium-ATPase pump dysfunction [32,33]. In this situation, we can assume that ATP-breakdowns reflects the state of the mitochondria. Therefore, the prevention of the ATP depletion observed in livers preserved with IGL-1 compared those preserved with UW and HTK seems to be consistent with an improved capacity of the mitochondria for recovery after the reperfusion insult, and consequently, with a better capacity for graft recovery.

Given that one of the functions of ALDH2 is to remove toxic adducts of this highly reactive aldehyde 4-HNE [19], the higher levels of ALDH2 protein that we see in the IGL-1 group when compared with UW and HTK (Figure 2a) may partially explain the reduction in hepatic damage observed in the IGL-1 group (Figure 1), even though the ALDH2 protein levels (almost twice as high as in the other groups) did not induce ALDH2 activity (Figure 2b).

The data reported here reveal a transient lipid peroxidation (Figure 3), which should be associated with the processes of organ recovery, manipulation, and preservation prior to its reperfusion. Reactive aldehydes, including MDA and 4-HNE, as well as advanced oxidation protein products (AOPP), have been found in almost all tissues undergoing ischemic injury [34]. Any of them might be the potential substrates of ALDH2. According to our results, the MDA and AOPP formations is more efficiently prevented by IGL-1 and HTK solutions than by UW (Figure 3a,b). ALDH2 did not have an impact on these values under our experimental conditions. By contrast, there was a close inverse correlation between the protein levels of ALDH2 and 4-HNE adducts (Figure 3c). The 4-HNE adducts inhibit key metabolic enzymes and aggravate the accumulation of damaged proteins [35]; in addition, 4-HNE is recognized as an early event in the pathogenesis of neurodegenerative diseases [36]. The fact that activation of ALDH2 decreases 4-HNE adducts has also been reported in stroke [37] and has been proposed as a strategy for treating or preventing ischemia.

The effective prevention of lipid peroxidation in IGL-1 cold stored fatty grafts was consistent with the upregulated expression of a well-known antioxidant and cytoprotective marker, Nfr2, against IRI [26] (Figure 4). The increased expression of Nrf2 in IGL-1-preserved grafts compared to UW and HTK seems to contribute to safeguarding the graft not only during ischemia [26], when some of these sub-products of oxidation are generated, but especially against those generated by the subsequent graft reperfusion and in later stages, after organ revascularization in transplantation procedures. Therefore, the induction of Nfr2 against cold ischemia injury in fatty livers seems to reduce the extent of the subsequent oxidative damage against reperfusion, in a step also called “preparation for oxidative stress” (POS) [38], and it is suggested that ALDH2 upregulates Nrf2 through the Nrf2/HO-1 antioxidant pathway [39]. This is of particular interest since HO-1 is overexpressed in fatty livers preserved in IGL-1 and then subjected to “ex vivo” perfusion [40]

ALDH2 activation was also consistent with the prevention of liver apoptosis (measured as caspase 3 activity levels and TUNEL assay findings) depending on the organ preservation solution used (Figure 5, Table 1).

IGL-1 and UW solutions have a similar composition, except for the oncotic agent used and the Na^+^/K^+^ ion reversal (see Table 1). We might suspect that the differences found in ALDH2/4-HNE levels may be associated with the presence/absence of the oncotic agent PEG35. This theory is supported by the fact that the presence or absence of PEG35-rinse solutions for liver grafts protects against mitochondrial damage [41,42], and by the comparison of IGL-1 (with PEG35) vs. HTK (without PEG35) in fatty liver graft preservation [43]. Moreover, recent preliminary research comparing IGL-1 with the same solution without oncotic agent PEG35 (IGL-0) found that the absence of PEG35 significantly reduced the fatty liver graft protection against ischemic insult after 24 h at 4 °C [44].

In conclusion, we found that the protective benefits of ALDH2 in the fatty liver grafts’ cold preservation depend on the organ preservation solution used, and were higher in IGL-1 than in UW and HTK. ALDH2 acts by cleansing the 4-HNE toxic adducts and improving the energetic balance of the cell (and thus, the mitochondrial status). ALDH2 helps to reduce the vulnerability of the fatty liver graft against ischemic insult, increases its ability to endure cold graft preservation and improves protection against the subsequent reperfusion damage, all of which favor the graft outcome after liver transplantation. Our data suggest that ALDH2 can be used as a therapeutic target to increase hepatoprotection, especially when sub-optimal livers for transplantation are used. So ALDH2 activators such as Alda-1 have a strong potential for use as additives for improving preservation solutions in situations of ischemia reperfusion injury.

## 4. Materials and Methods

### 4.1. Animals

Homozygous (obese (Ob)) Zücker rats aged 16–18 weeks from the were used. All animals had free access to water and dry food. All animals were used in accordance with protocols approved on 14 July 2016 (483116). The study was performed in accordance with the European Union Directive (EU guideline 86/609/EEC) for animal experiments and approved by the Ethics Committees for Animal Experimentation of the University of Barcelona (Directive 483/16). Animals were randomly distributed into groups as described below.

### 4.2. Experimental Groups

Protocol 1: In order to evaluate the ALDH2 activity/protein expression changes in the different organ preservation solution we considered the following experimental groups, as follows.

Group 1 (IGL-1; *n* = 6): After organ recovery, the livers were flushed with 50 mL of IGL-1 solution and stored in IGL-1 preservation solution for 24 h at 4 °C.

Group 2 (UW; *n* = 6): After organ recovery, the livers were flushed with 50 mL of UW solution and stored in IGL-1 preservation solution for 24 h at 4 °C.

Group 3 (HTK; *n* = 6): After organ recovery, the livers were flushed with 125 mL of HTK solution (2.5 times more than IGL-1) and stored in HTK solution for 24 h at 4 °C.

Group 4 (SHAM; *n* = 6): Animals underwent a transverse laparotomy and received silk ligatures in the right suprarenal vein, diaphragmatic vein, and hepatic artery. Samples were taken immediately afterward.

After cold storage, the liver specimens in the IGL-1, UW, and HTK solutions were washed with Ringer lactate solution (20 mL) and the samples were taken from the flush. The liver samples were then stored at −80 °C for the subsequent biochemical determinations.

### 4.3. Biochemical Determinations

#### 4.3.1. Transaminase Assay

Hepatic injuries was evaluated by alanine aminotransferase (ALT) and aspartate aminotransferase (AST) levels using commercial kits from RAL (Barcelona, Spain), as previously reported [30]. Briefly, 100 μL of effluent washout liquid was added to 1 mL of the substrate provided by the commercial kit, and transaminase activity was then measured at 340 nm with a UV spectrometer and calculated following the supplier’s instructions. Results were normalized using a commercial calibrator Biocal, RAL (Barcelona, Spain).

#### 4.3.2. Histology

Liver specimens were fixed in 10% buffered formalin solution and embedded in paraplast. Sections were made at 4 μm and stained with hemotoxylin-eosin. The histological severity of injury was graded on a scale from 0 (no lesion) to 4 (severe cellular damage such as cell dissociation, cell swelling, and disintegration of hepatic architecture).

#### 4.3.3. ALDH2 Activity

ALDH2 activity was assessed using an ALDH2 activity assay (Abcam Inc., Cambridge, UK ref.: ab115348) following the manufacturer’s instructions. ALDH2 activity measurements were carried out at 340 nm and expressed as NAD micromols /min per mg/protein.

#### 4.3.4. 4-Hydroxynonenal

Hydroxynonenal (HNE) protein adducts were measured in liver homogenate using the OxiSelect™ HNE Adduct Competitive ELISA Kit (Cell Biolabs, Inc. San Diego, CA, USA). The liver was homogenized in 10% (*w*/*v*) with a teflon bar in a RIPA solution, (Tris 50 M pH 7,4, 1% Triton 100×, NaCl 150 mM, NaF 5 M, 0.1% sodium dodecyl sulphate and 1% sodium deoxycholate) with an antiprotease solution (aprotinin at 1.7 mg/mL, 2 µg/mL pepstatin, 2 µg/mL leupeptin and 1 mM phenylmethylsulfonyl fluoride and sodium ortovanadate at 1 mM). The suspension was centrifuged at 2000× *g* for 5 min and the pellet discarded. Liver homogenates were added to a HNE conjugate preabsorbed ELISA plate. After a brief incubation, an anti-HNE polyclonal antibody was added, followed by an HRP conjugated secondary antibody. The quantity of the HNE adduct in protein samples was determined by comparing its absorbance with that of a known HNE-BSA standard curve.

#### 4.3.5. Malondialdehyde

Malondialdehyde (MDA), one of the end-products of lipid peroxidation, was determined by thiobarbituric reactive substances (TBARS) assay [1] in livers previously homogenated in RIPA (as described above). The formation of MDA-TBA adduct was fluorometrically measured at an excitation wavelength of 515 nm and an emission wavelength of 550 nm. The calibration curve was determined using tetraethoxypropane. Values are expressed as TBARS in nmol/mg protein.

#### 4.3.6. Advanced Oxidation Protein Products

Advanced oxidation protein products (AOPP) have been identified as a biomarker of oxidative damage to proteins, detecting dityrosine-containing and cross-linking protein products [2]. The formation of AOPP in the liver homogenates was spectrophotometrically measured at 340 nm. The results were obtained through a standard calibration curve using 100 μL of chloramine-T solution (0–100 μmol/L). The AOPP concentration was expressed in nmol/ mg protein.

#### 4.3.7. Caspase 3 Activity

Caspase activity was measured in liver homogenate using a commercial kit (CASP3C, Sigma-Aldrich, Madrid, Spain) according to the manufacturer’s instructions.

#### 4.3.8. ATP Measurements

The determination of ATP in liver samples homogenated in a perchloric acid solution was performed using the ATP assay kit for fluorimetry (Sigma Aldrich ATP colorimetric/fluorimetric assay kit, Madrid, Spain). The ATP concentration was determined by the phosphorylation of glycerol, a product that can be detected by the fluorimeter (excitation/emission 535/587 nm) at 37 °C and proportional to the amount of ATP in the sample.

#### 4.3.9. Western-Blotting Analysis of ALDH2, 4-HNE, Caspase 3 and Caspase 3 Activity

Separated on 6–15% sodium dodecyl sulfate polyacrylamide gel electrophoresis (SDS-PAGE) gels, proteins were blotted into poly-vinylidene fluoride (PVDF) membranes (Biorad, Madrid, Spain) and immunoblotted overnight at 4 °C using antibodies against: ALDH2 (Abcam Inc.), 4-HNE conjugates (NB100-63093, Novus Biologicals Europe, Abingdon, UK), and Nrf2 (sc-365949), Caspases 3 (Santa Cruz Biotechnology, Santa Cruz, CA, USA), Detection was performed with anti-IgG-HRP (Santa Cruz Biotechnology, Inc., Heidelberg, Germany). In all cases, the chemiluminescence signals were quantified by scanning using a GS800 calibrated densitometer (BioRad, Madrid, Spain). Both β-actin and α-tubulin were used as loading controls.

#### 4.3.10. TUNEL Assay

DNA fragmentation was determined using a TUNEL assay kit according to the manufacturer’s instructions (Apoptag Peroxidase In Situ Apoptosis Detection Kit, Merk, Darmstadt, Germany). Hepatic cells were analyzed in three different tissue sections per animal, and a total of 10 randomized HPF per animal were counted (400 hepatic cells per field). The ratio of positive cells to the total number was calculated.

### 4.4. Statistics

Data were expressed as the mean ± standard error, and compared statistically by variance analysis, followed by the Student–Newman–Keuls test using GraphPad Prism version 4.02 for Windows (GraphPad Prism software). *p* < 0.05 was considered significant.

## Figures and Tables

**Table 1:** The comparison between the University of Wisconsin (UW) solution vs. the histidine-tryptophan-ketoglutarate solution and Institut Georges Lopez (IGL-1) solutions.

	Component	IGL-1	UW	HTK
Colloids (mmol/L)	PEG35	0.03		
	HES		0.25	
Antioxidants (mmol/L)	Glutathione	3	3	
Precursors (mmol/L)	Adenosine	5	5	
	Ketoglutarate			1
Buffers (mmol/L)	Diphosphate	25	25	
	Histidine			198
	Histidine-HCl			18
	Tryptophan			2
Electrolytes (mmol/L)	K^+^	25	125	10
	Na^+^	120	27	15
	Mg^2+^		5	4
	Cl^−^			50
	SO_4_^2−^	5	4	
	Ca^2+^	0.5		0.015
Impermeants (mmol/L)	Raffinose	30	30	
	Lactobionic acid	100	105	
	Mannitol			30
	Osmolarity (mOs mol/l)	290	320	310
